# Key community eye health messages

**Published:** 2018-07-31

## Why invest in human resources for eye health?

**Figure:**
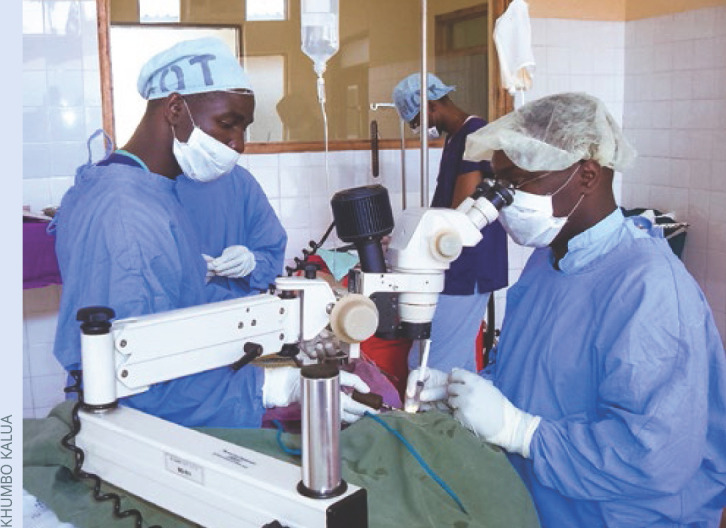

- A strong eye health workforce is essential if we are to achieve the goal of universal eye health for all. Strategic investment is needed to meet the growing demand for eye health services worldwide
- A shift in thinking is needed: spending money to train, recruit and keep eye health workers in post (retain them) is an investment not a cost

## What are the priorities for investment?

**Figure:**
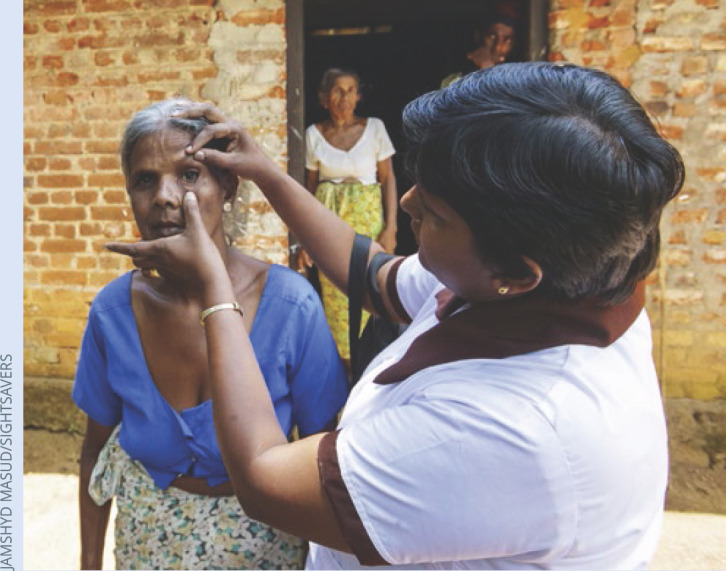

- Designing competency-based curriculums that allow trainees to develop essential skills
- Offering life-long learning opportunities/ continuing professional development
- Training allied opthalmic personnel in order to strengthen eye teams
- Creating and funding posts for skilled eye care workers, where they are needed
- Establishing well-functioning health facilities and offering appropriate incentives to help retain staff members in remote and rural areas

## How can we work strategically?

**Figure:**
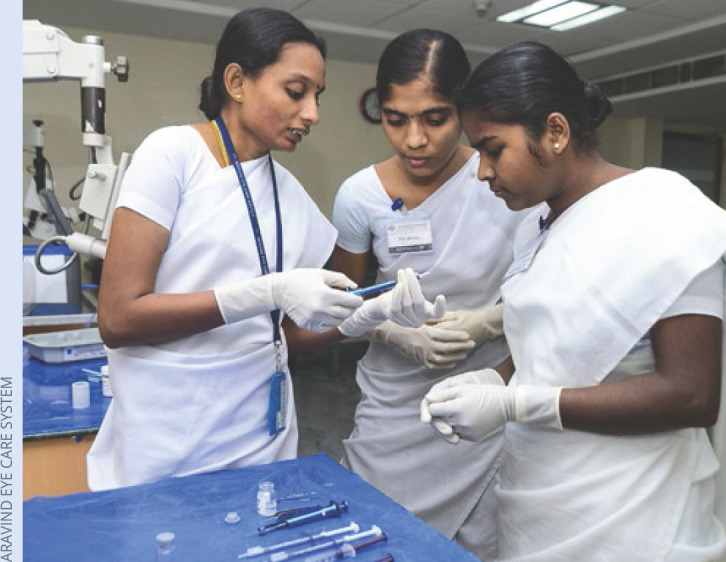

- Joined-up education and eye health workforce planning will help to ensure that there are enough trained people, with the right skills, to provide services where they are needed
- Investigate, research, and/or forecast local eye needs by volume and type of service needed. This will guide the training and placement of eye health workers

